# A Randomized controlled trial comparing patellar retention versus patellar resurfacing in primary total knee arthroplasty: 5–10 year follow-up

**DOI:** 10.1186/1756-0500-5-273

**Published:** 2012-06-07

**Authors:** Lauren Beaupre, Charles Secretan, DWC Johnston, Guy Lavoie

**Affiliations:** 1 2–50 Corbett Hall, University of Alberta, Edmonton, AB, T6G 2 G4, Canada; 2 2D2 Walter Mackenzie Centre, 8440-112 St, Edmonton, AB, T6G 2B7, Canada

**Keywords:** Total Knee Arthroplasty, Patellar Resurfacing, Revision, Patient-Reported Outcomes, Recovery

## Abstract

**Background:**

The primary purpose of this randomized controlled trial (RCT) was to compare knee-specific outcomes (stiffness, pain, function) between patellar retention and resurfacing up to 10 years after primary total knee arthroplasty (TKA). Secondarily, we compared re-operation rates.

**Methods:**

38 subjects with non-inflammatory arthritis were randomized at primary TKA surgery to receive patellar resurfacing (n = 21; Resurfaced group) or to retain their native patella (n = 17; Non-resurfaced group). Evaluations were performed preoperatively, one, five and 10 years postoperatively by an evaluator who was blinded to group allocation. Self-reported knee-specific stiffness, pain and function, the primary outcomes, were measured by the Western Ontario McMaster Osteoarthritis Index (WOMAC). Revision rate was determined at each evaluation and through hospital record review.

**Results:**

30 (88%) and 23 (72%) of available subjects completed the five and 10-year review respectively. Knee-specific scores continued to improve for both groups over the 10-years, despite diminishing overall health with no significant group differences seen. All revisions occurred within five years of surgery (three Non-resurfaced subjects; one Resurfaced subject) (p = 0.31). Two revisions in the Non-resurfaced group were due to persistent anterior knee pain.

**Conclusions:**

We found no differences in knee-specific results between groups at 5–10 years postoperatively. The Non-resurfaced group had two revisions due to anterior knee pain similar to rates reported in other studies. Knee-specific results provide useful postoperative information and should be used in future studies comparing patellar management strategies.

**ClinicalTrials.gov identifier:**

NCT01500252

## Background

Despite the success of total knee arthroplasty (TKA), surgeons are still seeking definitive indications for patellar resurfacing at the index surgery. Three approaches are currently used – 1) Never resurface, 2) Always resurface, and 3) Selectively resurface the patella [1]. Unfortunately, systematic reviews, meta-analyses [1-7] and randomized clinical trials (RCTs) [8-13] have been unable to demonstrate clear superiority of one approach over the other.

Current evidence is limited by study heterogeneity (devices/manufacturers/patients/outcomes evaluated) and limited follow-up of less than five years. In studies with early to mid-term follow-up, there appears to be an increased reoperation rate within five years due to ongoing anterior knee pain when patellar resurfacing is not performed [3-5]. The findings regarding anterior knee pain have been mixed, with a trend towards supporting patellar resurfacing [3-6]. However similar satisfaction and functional outcomes have been reported among both groups [4,14].

In the few longer-term studies, [9,15-17] both groups appear to maintain similar outcomes, but typically there is high subject attrition. Further, most studies have only used performance-based tests and general function scores, but these outcomes are affected by subject aging and overall diminishing health status [15-17]. Only one long-term study has examined patient-reported knee-specific pain, function and stiffness, which are less likely to be affected by overall health changes [9].

The primary study objective was to compare pain, stiffness and function over the first 10 postoperative years using a disease-specific index between subjects receiving patellar resurfacing (Resurfacing group) and those who retain their patella (Non-resurfaced group) at primary TKA. The secondary objective was to compare revision rates and general health status over 10 years.

## Methods

### Study design

We performed a parallel design double-blind RCT comparing patellar resurfacing versus patellar retention. Randomization sequence was computer-generated in blocks of 10 subjects with randomization codes stored in sequentially numbered opaque envelopes that were opened in the operating room. Ethical approval was obtained from the Health Research Ethics Board – Biomedical Panel at the University of Alberta, initially for the five years follow-up, but was extended to allow longer-term follow-up (Pro00002794). All subjects were competent to provide consent and signed informed consent forms prior to the initial assessment. The authors have no potential or known conflicts of interest to claim for this work.

Power analysis was based on a clinical estimation that 20 points on the Western Ontario McMaster Osteoarthritis Index (WOMAC) would represent a clinically important difference [16,18]. Thus, 14 patients per group were required; 38 patients were randomized to account for patient attrition. We had five randomization blocks because subjects were randomized after their initial clinical assessment and occasionally surgeons excluded them at surgery because the study implant was deemed unsuitable (n = 6). The original study timeline was five-years postoperatively, but was extended to 10-years to provide longer-term results.

### Selection criteria

Subjects were recruited from 1996 to 1999 from three fellowship-trained arthroplasty surgeons at one tertiary Canadian health center during their preoperative assessment. Eligible subjects were scheduled for primary TKA to treat non-inflammatory arthritis and were less than 75 years old. Subjects were excluded if they had a history of knee sepsis, previous patellectomy, high tibial osteotomy, knee flexion contracture, varus/valgus deformity of greater than 20 degrees, less than 90 degrees of knee flexion or tibial or femoral bone deficiency requiring augmentation.

### Intervention

The Profix™ Total Knee System, a posterior cruciate retaining, fixed bearing prosthesis manufactured by Smith and Nephew, Inc. was utilized in all subjects. Subjects randomized to the Resurfaced group received an all polyethylene patellar implant while those randomized to the Non-resurfaced group had no operative intervention involving the patella. Standard surgical technique including a midline incision and medial parapatellar exposure was utilized and all components were cemented. All surgeries were done under tourniquet and a postoperative drain was utilized.

A standardized clinical pathway was followed ensuring that all subjects received similar preoperative, perioperative and postoperative care; early mobilization was encouraged starting the first postoperative day. All subjects were weightbearing as tolerated with the assistance of walking aids for the first six postoperative weeks.

### Evaluation

Subjects completed the WOMAC and RAND-36 questionnaires and a physical therapist assessed their knee range of motion (ROM) preoperatively. Comorbidities were also collected on admission to the study. The WOMAC questions were directed specifically to their operative knee. Subjects were randomized after the assessment, thereby blinding the evaluator to group allocation. Subjects were also blinded to group allocation.

Subjects were re-assessed independent of the surgeon at one and five years postoperatively by physical therapists not involved in subjects’ care who were blinded to group allocation. They completed the same questionnaires and were asked about complications and re-operations. Ten-years postoperatively, subjects completed the questionnaires by telephone and were also asked about further re-operations on their knee. The WOMAC Index questions were, again, directed specifically to their operative knee. Regional record reviews were undertaken for all subjects alive at 10-years regardless of whether or not they completed the telephone interview to ensure that re-operations were not missed.

### Outcomes

The primary outcomes were knee-specific patient-reported WOMAC scores. Secondary outcomes were revisions, defined as knee re-operation for any reason within the first 10 postoperative years, and general health status as measured by the RAND-36.

The WOMAC, a reliable, valid and responsive scale for TKA patients, was used to determine patient-reported joint-specific pain, stiffness and function [19,20]. We used the initial version of the WOMAC [21,22]. Each WOMAC subscale score was transformed from zero to one hundred points, with a score of 100 indicating no pain or dysfunction, so that the WOMAC and RAND-36 scores were unidirectional [23]. In recent years, clinically important differences on the WOMAC measured as changes in scores [16,18,24] or effect sizes [16,18,24] have been reported. However, this information was not available at the time of study commencement. Further, it is important to note that the WOMAC stiffness index measures how stiff the knee was upon a) first waking in the morning and b) after sitting for prolonged periods and was not considered a proxy measurement of knee ROM.

The RAND-36 was used to determine overall general health status. The RAND-36 is a reliable and valid generic health status questionnaire for TKA populations that contains identical items as the Short Form-36 (SF-36) [19,20,23,25]. We focused on the General Health dimension, which ranges from 0–100 where a higher score indicates better health.

Knee ROM was followed to one-year and not considered a formal study outcome. Acceptable ROM was achieved with average knee flexion of 98.6 ± 10.4 in the Resurfaced group and 98.6 ± 11.2 in the Non-resurfaced group (p = 1.00) at one year postoperatively.

### Analysis

All analyses were performed “intention to treat”, in which subjects’ data are analyzed as per group allocation. Descriptive statistics (means, standard deviations and proportions) were generated for all study variables.

As a measure to balance the preoperative WOMAC stiffness differences between groups, we evaluated the change in WOMAC indices and the RAND general health status between each measurement period using independent t-tests. Revision and complication rates were compared between groups using non-parametric Chi square or Fisher’s Exact tests.

All analyses were performed with the Statistical Package for the Social Sciences (SPSS) version 17.0 (SPSS Inc., Chicago, Illinois, USA) utilizing two-tailed tests. A significance level of p ≤ 0.01 was utilized because multiple comparisons were made.

## Results

### Baseline characteristics

Thirty-eight subjects were enrolled with 21 randomly allocated to the Resurfaced and 17 to the Non-resurfaced group. The groups were similar in baseline characteristics with the exception of WOMAC stiffness, where the Resurfaced group reported worse preoperative stiffness on the WOMAC stiffness indices (Table 1).

**Table 1:** Baseline characteristics of 38 patients randomized to receive patellar resurfacing or non-resurfacing in primary total knee arthroplasty

	**Resurfaced**	**Non-resurfaced**	**P value**
**Demographics**			
**Mean Age in yr (SD)**	64.9 (4.0)	62.0 (5.6)	0.07^*^
**Females (%)**	16 (76)	10 (59)	0.31^†^
**BMI (SD)**	29.8 (5.9)	33.9 (7.3)	0.10^*^
**0-1 Comorbidities (%)**	17 (85)	12 (71)	0.43^†^
**Baseline characteristics**			
**WOMAC score**			
** Stiffness (SD)**	31.5 (10.9)	46.9 (21.7)	0.01^*^
** Pain (SD)**	38.1 (10.8)	43.8 (10.2)	0.12^*^
** Function (SD)**	42.7 (9.9)	47.7 (12.7)	0.20^*^
**RAND 36 score**			
** Physical Functioning (SD)**	20.6 (10.9)	31.7 (21.9)	0.06^*^
** Role Physical (SD)**	7.1 (17.9)	18.7 (33.5)	0.18^*^
** Bodily Pain (SD)**	30.6 (12.2)	38.9 (17.0)	0.09^*^
** General Health (SD)**	70.5 (18.0)	71.8 (18.4)	0.83^*^
**Range of Motion in degrees (SD)**	107.9 ± 15.6	112.0 ± 9.8	0.23^*^

LEGEND: SD = Standard Deviation.

^*^ Analyzed using an Independent *T*-test.

^†^ Analyzed using Fisher’s Exact Test.

### Follow-up

**Figure 1:**
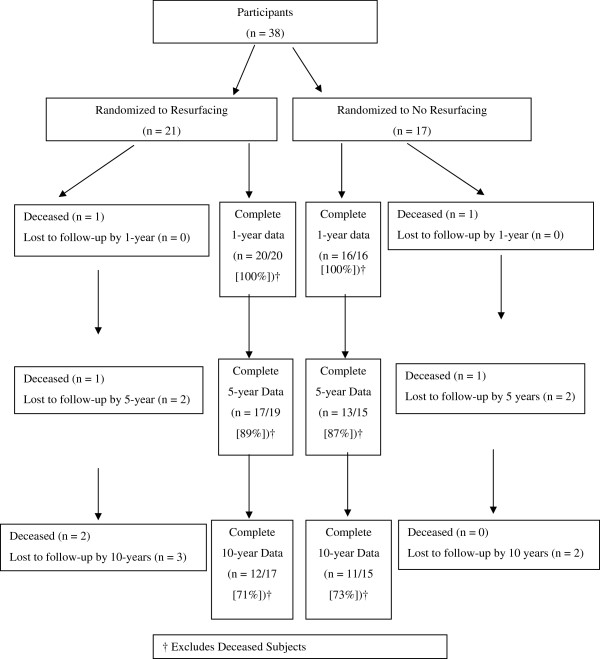
Flowchart of Subjects in the Trial.

Six subjects (Resurfaced = 4, Non-resurfaced = 2; [p = 0.63]) died within 10 years due to conditions unrelated to their TKA. Of those available, 30 (88%) were followed to five years and 23 (72%) to 10 years, with similar losses to follow-up between groups (Resurfaced = 5, Non-resurfaced = 4; [p = 1.00]) (Figure 1).

## Womac

### Stiffness

Although baseline differences were noted between groups, with the Resurfaced group reporting significantly more preoperative stiffness, the groups reported clinically similar stiffness scores at one-year. When group comparisons were made of change in stiffness between each evaluation, these differences did not attain statistical significance (Table 2). Neither group reported deterioration in knee-specific stiffness between five and 10 years.

**Table 2:** Mean Change in WOMAC and RAND-36 General Health Between Measurement Periods

	**Resurfaced**	**Non-resurfaced**	P value*
**Stiffness**			
Mean Change from preop to 1 year postop (SD)	24.4 ± 24.5	8.3 ± 32.3	0.10
Mean Change from 1 year to 5 years postop (SD)	15.5 ± 22.1	16.3 ± 24.7	0.66
Mean Change from 5 years to 10 years postop (SD)	3.4 ± 33.6	15.0 ± 19.4	0.35
**Pain**			
Mean Change from preop to 1 year postop (SD)	32.9 ± 18.2	34.3 ± 21.5	0.83
Mean Change from 1 year to 5 years postop (SD)	3.8 ± 17.7	3.1 ± 22.3	0.93
Mean Change from 5 years to 10 years postop (SD)	12.7 ± 20.0	10.0 ± 12.0	0.71
**Function**			
Mean Change from preop to 1 year postop (SD)	24.1 ± 16.6	19.5 ± 16.9	0.44
Mean Change from 1 year to 5 years postop (SD)	−3.8 ± 15.9	5.5 ± 19.8	0.18
Mean Change from 5 years to 10 years postop (SD)	14.2 ± 17.7	14.4 ± 13.6	0.98
**RAND-36 General Health**			
Mean Change from preop to 1 year postop (SD)	−8.2 ± 17.5	5.8 ± 10.6	0.02
Mean Change from 1 year to 5 years postop (SD)	−3.7 ± 14.4	−9.0 ± 19.1	0.43
Mean Change from 5 years to 10 years postop (SD)	−6.0 ± 15.8	−3.8 ± 15.4	0.75

LEGEND: SD = Standard Deviation.

^*^ Analyzed using an Independent *T*-test.

### Pain

Both groups reported a significantly improved pain score when compared to the baseline over the 10 year evaluation with no group differences noted (Table 2). In fact, pain scores continued to improve over the entire study horizon. For the WOMAC question that specifically asked about pain while doing stairs, the Resurfaced group reported slightly worse pain than the Non-resurfaced group, but these differences were not significant at any time point (p > 0.18).

### Function

Parallel findings were seen with function; both groups demonstrated substantial limitations in self-reported function preoperatively that improved significantly by one year and were maintained at five-years postoperatively (Table 2). Despite increasing age, both groups reported higher knee-specific function at 10 years than at five years.

#### Revisions

Four subjects underwent TKA revision within 10 years, with no group differences noted (p = 0.31). Two Non-resurfaced subjects and one Resurfaced subject were revised within the first postoperative year; both revisions in the Non-resurfaced group were for persistent anterior knee pain. Consequent patellar resurfacing improved their symptoms. In both cases, the primary operative report noted that there was little patellar articular cartilage. The Resurfaced revision was for knee instability secondary to insufficient polyethylene liner thickness. A third Non-resurfaced subject required re-operation after two years for septic arthritis secondary to a perforated viscus, which resulted in hematogenous spread of enterococcus to the TKA. No further revisions were reported after five-years.

### RAND-36 general health

RAND-36 General Health scores were similar between groups preoperatively (Table 1). General Health deteriorated slightly within five years and slightly further decreased at 10 years compared with the five-year results, suggesting decreasing overall health status over time (Table 2).

## Discussion

Despite meta-analyses, [1,5,26] systematic reviews [2-7] and RCTs, [8-13] the answer to whether or not the patella should be routinely resurfaced during primary TKA remains unanswered. Our study found, similar to others, subjects reported satisfactory long-term outcomes regardless of patellar management approach [4,14]. However, we also noted, similar to others, [5] that there is an increased re-operation rate due to persistent anterior knee pain in approximately 10% of subjects who do not undergo patellar resurfacing. The study subjects who experienced this phenomenon required patellar resurfacing relatively early in the postoperative period.

Our study would also suggest that long-term evaluation provides useful information. Subjects reported further improvements in knee-specific stiffness, pain and function between five and 10 years, which may be related to decreasing knee demands with increasing age or continued improvement in knee performance.

The few long-term studies available on patellar management strategies reported on performance-based outcome measures that subjects had difficulty completing at the 10-year evaluation due to limited physical health status [15-17]. We used patient-reported joint-specific outcomes that discriminated between the patients’ perception of the TKA performance and their overall health status. Patient-reported measures may be better for long-term assessment than performance-based outcomes, which can be attenuated by decreasing physical performance with increasing age. Campbell et al. [9] also found that there were few changes in knee specific outcomes between four and 10 years postoperatively. Use of these measures may also give a clearer indication of TKA outcomes than satisfaction questionnaires as satisfaction may reflect not only satisfaction with outcome, but also with care provided. Evaluators and subjects were also blinded to group allocation; thus our results likely do not reflect reporting bias.

Our study does have some major limitations. This was a small study using a single implant and we were likely under-powered based on current knowledge of important differences on the WOMAC Index, particularly stiffness. [16,18,24] In addition, we set the level of significance for the original power analysis at p < 0.05, but recognized at time of data analysis that we were making multiple comparisons and re-set the level of significance to p < 0.01 prior to starting the analysis. Further, we were also underpowered to detect differences in re-operation rates, an important surgical consideration. Although subjects reported similar results at 10-years, a 10% reoperation rate related to anterior knee pain within the first five postoperative years is not insignificant.

There were also stiffness differences between groups at baseline that remained at 10 years after TKA; thus it is possible that our reported results are related to these particular subjects and not patellar management. Stiffness has not commonly been evaluated in studies comparing patellar management strategies. Although related to knee ROM, stiffness does not necessarily mean that knee ROM was limited. [27] The WOMAC stiffness questions are related to initiating movement after sedentary periods rather than to overall knee movement. [19,20] More research is required to determine whether knee stiffness is related to patellar management.

In addition, we did not specifically measure anterior knee pain, but rather looked at how the overall TKA was performing. In examining the WOMAC pain question specifically asking about pain when performing stairs, the groups reported similar scores over the entire evaluation period. Patients also reported no major differences in overall functional outcomes, including stair climbing even when the function related specifically to their TKA.

Our study also focused on patient-reported outcomes rather than specific radiographic or surgical measures. Although patient-reported outcomes are important in determining how the patients were managing with either type of patellar management, our conclusions are limited to these measures rather than more direct surgical measures. Future studies should ensure that they measure both outcomes to get a full clinical picture of the impact of the different patellar management strategies.

Similar to the other long-term studies, we had high patient attrition by 10-years. [9,15-17] Our region is a large tertiary health region that provides arthroplasty care for most of the Northern half of our province in Canada, so these patients should receive further surgical care in our health region. Our health record check of patients not available at 10-years did not find further re-operations between five and 10 years postoperatively, but it is possible that some re-operations were missed.

## Conclusion

In summary, we found that approximately 10% of subjects retaining their native patella required patellar resurfacing early in the postoperative time period for persistent anterior knee pain. Overall, knee-specific score suggested that there were no major differences in functional outcomes over the 10-year evaluation and that TKA function was maintained over time even in the presence of diminishing general health status. These results would suggest that patient-reported and knee-specific outcomes provide useful additional information that performance-based measurement may lack over long-term evaluation. Unfortunately, although we have determined additional factors to consider and measure in future studies, we cannot make any conclusive statements regarding patellar management in primary TKA.

## Competing interests

This study was supported by an unrestricted research grant from Smith and Nephew INC.

## Authors’ contributions

Dr. Beaupre assisted with study design, data collection, data analysis and drafted the manuscript. Dr. Secretan assisted with data collection, data analysis and critical revision of the manuscript. Drs. Johnston and Lavoie assisted with study design, data collection, critical revision of the manuscript. All authors read and approved the final manuscript.

## Authors information

Dr. Beaupre receives salary support from Alberta Innovates- Health Solutions as a Population Health Investigator.
